# Change of Direction in the Biomechanics of Atherosclerosis

**DOI:** 10.1007/s10439-014-1095-4

**Published:** 2014-08-20

**Authors:** Yumnah Mohamied, Ethan M. Rowland, Emma L. Bailey, Spencer J. Sherwin, Martin A. Schwartz, Peter D. Weinberg

**Affiliations:** 1 Department of Bioengineering, Imperial College London, London, SW7 2AZ UK; 2 Department of Aeronautics, Imperial College London, London, UK; 3 Division of Cardiology, Department of Cell Biology, Yale University School of Medicine, New Haven, CT USA

**Keywords:** Hemodynamics, Wall shear stress, Atherosclerosis, Transverse wall shear stress, Oscillatory shear index, NF-κB, eNOS

## Abstract

The non-uniform distribution of atherosclerosis within the arterial system has been attributed to pro-atherogenic influences of low, oscillatory haemodynamic wall shear stress (WSS) on endothelial cells (EC). This theory is challenged by the changes in lesion location that occur with age in human and rabbit aortas. Furthermore, a number of point-wise comparisons of lesion prevalence and WSS have failed to support it. Here we investigate the hypothesis that multidirectional flow—characterized as the average magnitude of WSS components acting transversely to the mean vector (transWSS)—plays a key role. Maps of lesion prevalence around aortic branch ostia in immature and mature rabbits were compared with equivalent maps of time average WSS, the OSI (an index characterizing oscillatory flow) and transWSS, obtained from computational simulations; Spearman’s rank correlation coefficients were calculated for aggregated data and 95% confidence intervals were obtained by bootstrapping methods. Lesion prevalence correlated positively, strongly and significantly with transWSS at both ages. Correlations of lesion prevalence with the other shear metrics were not significant or were significantly lower than those obtained for transWSS. No correlation supported the low, oscillatory WSS theory. The data are consistent with the view that multidirectional near-wall flow is highly pro-atherogenic. Effects of multidirectional flow on EC, and methods for investigating them, are reviewed. The finding that oscillatory flow has pro-inflammatory effects when acting perpendicularly to the long axis of EC but anti-inflammatory effects when acting parallel to it may explain the stronger correlation of lesion prevalence with transWSS than with the OSI.

## Introduction

### Overview

A striking feature of atherosclerosis is its predilection for certain well-defined arterial sites, particularly within areas of branching and curvature. This propensity led to the suggestion that hemodynamic stresses are critical in atherogenesis, and has motivated extensive research into endothelial mechanotransducers and downstream signaling pathways. It has recently been argued that the directionality of the hemodynamic stresses is a key factor^26^ and, independently, that the direction of hemodynamic stresses can alter the balance of pro- and anti-atherosclerotic signals within endothelial cells (EC).^34^ Here we explain why this new biomechanical concept is required, present a quantitative study providing further evidence for a relation between flow directionality and disease, describe methods by which underlying mechanisms can be studied, and discuss relevant findings concerning cellular and molecular pathways.

### Historical Background

The systematic study of lesion location commenced with Anitschkow’s finding that aortic lipid deposits develop in an arrowhead-shaped region surrounding the downstream half of branch ostia in the cholesterol-fed rabbit.^1^ Anitschkow suggested that mechanical forces were involved, but it was Fry^14^ who proposed the more specific hypothesis that the formation of lesions downstream of branches in hyperlipidemic animals is caused by an elevation of haemodynamic wall shear stress (WSS) in such regions.

At around the same time, Caro *et al.* 8 were studying disease patterns in *post mortem* human aortas. Their model studies confirmed that areas downstream of branch mouths were exposed to high WSS but, contrary to the results of Anitschkow, they found that such areas had a particularly low frequency of disease. They consequently proposed that high WSS is protective and that disease is triggered instead by low WSS. Subsequently Ku *et al.*,^21^ by applying laser Doppler anemometry to transparent replicas of human carotid bifurcations, provided evidence that human atherosclerosis occurs in regions where near-wall flow is oscillatory. A new index, the Oscillatory Shear Index (OSI) was developed to capture this pattern of stresses. As later modified by He and Ku,^16^ it is defined as:

$${\text{OSI}} = \frac{1}{2}\left( {1 - \frac{{\left| {\int_{0}^{T} {\vec{\tau }_{w} dt} } \right|}}{{\int_{0}^{T} {\left| {\vec{\tau }_{w} } \right|dt} }}} \right) = \frac{1}{2}\left( {1 - \frac{{\left| {\vec{\tau }_{\text{mean}} } \right|}}{\text{TAWSS}}} \right)\,{\text{where}}\,\,\vec{\tau }_{\text{mean}} = \frac{1}{T}\int\limits_{0}^{T} {\vec{\tau }_{w} dt}$$

where $\vec{\tau }_{w}$ represents the instantaneous WSS vector, *t* the time, *T* the cardiac cycle and TAWSS the time average WSS. Because high OSI is associated with low time average WSS (the latter appearing in the denominator of the former), the theories of Caro *et al.* and of Ku *et al.* have become combined to some extent. The concept that low, oscillatory WSS triggers atherosclerosis underlies most current research concerning localizing factors.

### Several Patterns of Lesions Occur in Human Arteries

It is not obvious how the low, oscillatory WSS theory could account for the pattern of atherosclerosis observed by Anitschkow and subsequently confirmed by many others. That might appear unimportant; it is certainly conceivable that lesions in hyperlipidemic rabbits have different localizing factors from those involved in human disease. Indeed, Caro *et al.* 8 suggested that entry of cholesterol from blood into the wall might be critical in hyperlipidemia whilst exit into the lumen of lipids made or modified in the wall might be limiting in normolipidemia, leading to mirror image patterns of disease. We have suggested, however, that the different patterns reflect a difference in age.^37^ The apparent discrepancy, it is proposed, results from an inappropriate comparison of *immature* animal vessels with *mature* human ones. (Young rabbits are generally used for reasons of cost, whereas *post mortem* human tissue usually derives from adult cadavers).

In support of this idea, we have shown that the area downstream of branches is spared when mature rabbits are administered a cholesterol-rich diet.^4^ The fact that immature and mature rabbits develop different patterns of disease despite being on the same diet for the same length of time^10^ argues against the idea that the pattern depends on the relative levels of lipid in the blood and the wall. Evidence that there is a parallel change with age in human lesion patterns is provided by the work of Sinzinger *et al*.,^32^ who observed lipid deposition downstream of branch ostia in the aortas of human fetuses, newborns and infants; the pattern resembled the one observed by Anitschkow in immature rabbits and not the one observed by Caro *et al.* in mature human aortas. Additional age-related changes in human aortas were demonstrated by Sloop *et al.* 33: lesions occurred at the lateral margins of branch ostia in early adulthood, but upstream of ostia later on.

### A Re-evaluation of the Low, Oscillatory WSS Theory

The occurrence of several patterns of lesions in human (and rabbit) arteries provides an obvious challenge to the low, oscillatory WSS theory. Are all patterns of lipid deposition explained by low, oscillatory WSS, requiring that it occurs at different sites at different ages? Or is there a change with age in the type of stress that triggers lesions?

We investigated this question by comparing maps of lesions with maps of WSS (derived from computational simulations of steady flow) in immature and mature rabbit aortas.^24^ Both data sets were derived from 4 to 9 rabbits at each age, both were mapped at high resolution, and the comparison was made using a novel statistical method that did not assume a linear relationship between the variables or independence of neighboring grid squares in each map. Assessments were made separately for the proximal segment and the middle segment of the descending thoracic aorta and for regions around intercostal branch ostia.

Patterns of WSS, like patterns of lesion prevalence, change with age in the rabbit.^25^ That appears to be caused by a change in the amount of taper of the aorta, which alters the persistence down the vessel of Dean vortices generated in the arch.^25^ Despite both patterns changing with age, the evidence for a correlation between them was not strong^24^: significance was obtained for the proximal descending thoracic aorta at both ages, but not for the middle segment at either age. Around branch ostia, the correlation was borderline in immature animals and not significant in mature ones. Even where significance was obtained, the relation was always positive; that is, high lesion frequencies were associated with high WSS—no evidence was obtained for an association with low WSS in any location at either age.

The failure to obtain any evidence for the low shear stress theory, despite examining mature rabbits which—at least in the vicinity of branches—have a lesion pattern resembling one found in adult human aortas, led us to re-examine the strength of the published data for the theory. A systematic review was conducted,^27^ examining all papers in PubMed that contained the following search terms: atherosclerosis (or a variety of words describing the same disease), shear, and one of 15 words indicating that shear stresses had been obtained by computational fluid dynamics. The search was restricted to numerical studies because experimentally derived WSS is currently less reliable. The resulting 406 articles were then evaluated against a set of pre-defined inclusion and exclusion criteria, leaving 40 studies. Of these, 32 (80%) were thought by their authors to support the low, oscillatory WSS theory.

Superficially, that seemed like good evidence for the theory. However, a more detailed examination presented a different picture. Studies were ranked in categories depending on the degree of quantification and data reduction used when making the comparison between shear and disease; those in the lowest category presented a purely descriptive analysis and those in the highest category employed a point-by-point statistical comparison.^27^ When subdivided in this way, the 27 studies in the lowest four categories universally supported the low, oscillatory WSS theory, the eight studies in the next two categories (which made quantitative, spatially resolved comparisons between shear and disease metrics but used either axial or circumferential averaging) were approximately evenly divided, and all five studies in the most rigorous category failed to support the theory.

### Development and Preliminary Evaluation of a New WSS Metric

**Figure 1:**
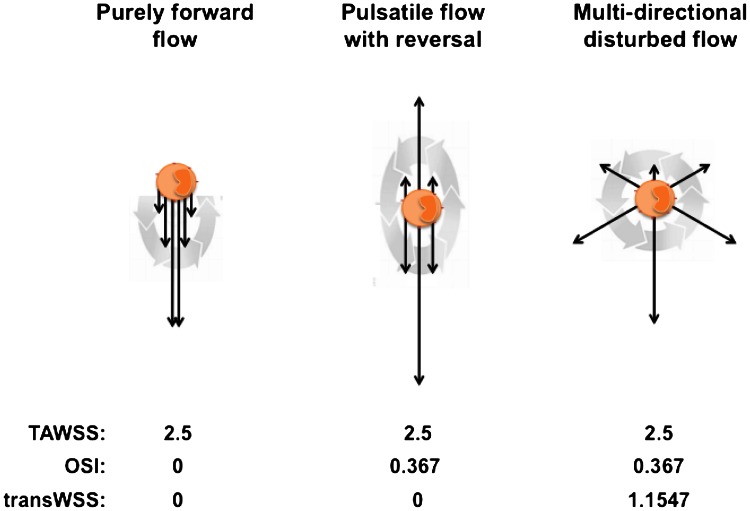
Three different flow environments to which an EC could be exposed. The black arrows represent WSS vectors at various times in the cardiac cycle. The gray arrows indicate their evolution with time during the cardiac cycle. The table lists TAWSS (Pa), OSI, and transWSS (Pa) for the three environments. Modified from Ref. 26

We speculated that a different metric of so-called “disturbed flow,” perhaps associated with different influences on the biology of EC, might give a better correlation.^26^ Examination of flows and cellular responses in experiments where EC were cultured in swirling dishes, described in more detail below, led us to suggest that multidirectional WSS might be particularly atherogenic. The OSI does not distinguish well between uniaxial pulsatile flow and multidirectional flow. Figure 1 shows that it is possible to obtain the same time averaged WSS and the same OSI for two very different flow regimes, one consisting of purely uniaxial near-wall flow and the other being truly multidirectional. To distinguish between them, we developed a new metric, the transverse WSS (transWSS), to capture multidirectionality.^26^

The metric is defined as:

$${\text{transWSS}} = \frac{1}{T}\int\limits_{0}^{T} {\left| {\vec{\tau }_{\omega } \cdot \left( {\vec{n} \times \frac{{\int_{0}^{T} {\vec{\tau }_{\omega } dt} }}{{\left| {\int_{0}^{T} {\vec{\tau }_{\omega } dt} } \right|}}} \right)} \right|} dt$$

where $\vec{n}$ represents the normal to the arterial surface. It is equivalent to:

$${\text{transWSS}} = \frac{1}{T}\int\limits_{0}^{T} {\left| {\vec{\tau }_{\omega } \cdot \left( {\vec{n} \times \frac{{\vec{\tau }_{\text{mean}} }}{{\left| {\vec{\tau }_{\text{mean}} } \right|}}} \right)} \right|} dt$$

and it averages over the cardiac cycle the magnitude of those components of the instantaneous WSS vector that are perpendicular to the mean WSS vector in the plane of the endothelium.

TransWSS by itself does not completely characterize the pattern of WSS. For example, it cannot distinguish between unidirectional flow and reversing but uniaxial flow, both of which have a transWSS of zero (Fig. 1); the OSI and time average WSS are still required to capture these potentially important aspects of flow dynamics. However, transWSS does distinguish between uniaxial and multidirectional flows even when they have the same OSI. There are some limitations to the applicability of transWSS but they appear unlikely to be important in practice, and the metric has clear advantages over others that have attempted to capture aspects of multidirectionality—these points are reviewed elsewhere.^26^

### Preliminary Results and Need for a Further Study

When the metric was used to post-process simulations of pulsatile flow in immature and mature rabbit aortas, patterns of transWSS were observed at some branches that showed a remarkable resemblance to patterns of diet-induced lipid deposition at the two ages.^26^ However, the results of our systematic review^27^ discourage subjective comparisons between patterns of WSS metrics and disease. Here we present a new study that quantified the correlation and compared it with those obtained for time average WSS and the OSI.

## Methods

The investigation employed novel statistical techniques to compare average maps of WSS metrics and lesion frequencies around intercostal branch ostia in immature and mature rabbits. The WSS and lesion data derived from two previous studies^10^,^26^ that are only briefly summarized here.

### Computational Fluid Dynamics Study

Simulations of pulsatile flow in the thoracic aortas of two immature and two mature male New Zealand White rabbits (HSDIF strain) were conducted using the in-house spectral/*hp* element solver Nεκταr.^26^ Aortic geometries were obtained from micro-CT scans (nominal 50-*μ*m resolution) of vascular corrosion casts.^25^ The casts may have been affected by resin shrinkage during polymerisation but WSS maps appear insensitive to precise intercostal branch geometry.^19^ The aortic inflow velocity profile was blunt; an earlier parametric study^25^ showed that flow solutions are insensitive to this assumption too. The inflow waveform was physiological with a mean Reynolds number of 300. Branch outflow waveforms had the same shape as the aortic waveform, as suggested by the Doppler ultrasound data of Sloop *et al.*,^33^ and were in phase with it. Approximately 0.2% of aortic flow entered each intercostal artery, consistent with the velocity data of Sloop *et al.* 33 and the diameter ratios of the two vessels. (Although the *magnitude* of WSS variations around ostia is sensitive to branch flow rate, the character of the pattern is not affected to the same extent.^19^) A zero velocity gradient/constant pressure boundary condition was imposed at the extended aortic outflow. Walls were rigid, which again appears to make little difference to WSS results [data not shown]. Tethering, and therefore asymmetric movement of the aorta during the cardiac cycle, was ignored; Zeng *et al*.^39^ have demonstrated that this is a reasonable approximation even for the coronary arteries.

### Disease Localisation

Aortic lipid deposition was induced in 8 immature and 9 mature male New Zealand White rabbits of the HSDIF strain by administering a diet supplemented with 1% cholesterol for 8 weeks.^10^ Lesions were stained with oil red O and, after counterstaining the aorta with Evans’ Blue Dye, were imaged *en face* at a nominal resolution of 8 *μ*m using a flatbed scanner. A grid with line spacing equivalent to 120 *μ*m was superimposed on each image so that the presence or absence of lesions could be manually scored in each square, and frequency-of-occurrence (“prevalence”) maps were obtained by combining the maps.^9^

### Analysis of the Maps

The study analyzed maps centred on intercostal branch ostia in the proximal and middle segments of the descending thoracic aorta. The maps had a size equivalent to 1.92 × 1.92 mm in immature rabbits and 2.4 × 2.4 mm in mature ones, approximately compensating for the change of size with age. They were obtained by averaging data from 112 immature and 126 mature branches examined in the lesion study,^10^ and from 18 immature and 20 mature branches in the numerical study.^26^

Each haemodynamic metric was correlated with lesion prevalence at each age, using Spearman’s rank coefficients to avoid the assumption of a linear relation. To avoid the problem of autocorrelation between grid squares, confidence intervals of the coefficients were assessed using a bootstrapping approach. Briefly, many additional lesion prevalence maps were created by repeatedly drawing new samples from the original data; the same number of individual branch maps was used in each of these samples as when creating the original average map, but because sampling with replacement was used, some branches would have been included more than once and others not at all. Repetition of the process gives an estimate of the sampling distribution of the original map. The same procedure was carried out for the shear metrics, and correlation coefficients were then computed for different pairwise comparisons of the shear and lesion maps, leading to a distribution of correlation coefficients from which a confidence interval was obtained. (Further details of the statistical methods will be presented elsewhere.) The significance of the correlation coefficients was determined by observing whether the confidence interval included a coefficient of zero, and the significance of differences between coefficients was determined by observing whether their absolute confidence intervals overlapped. (It was necessary to consider only magnitudes, and to ignore signs, so that the strength of a positive correlation could be compared with the strength of a negative one).

## Results

**Figure 2:**
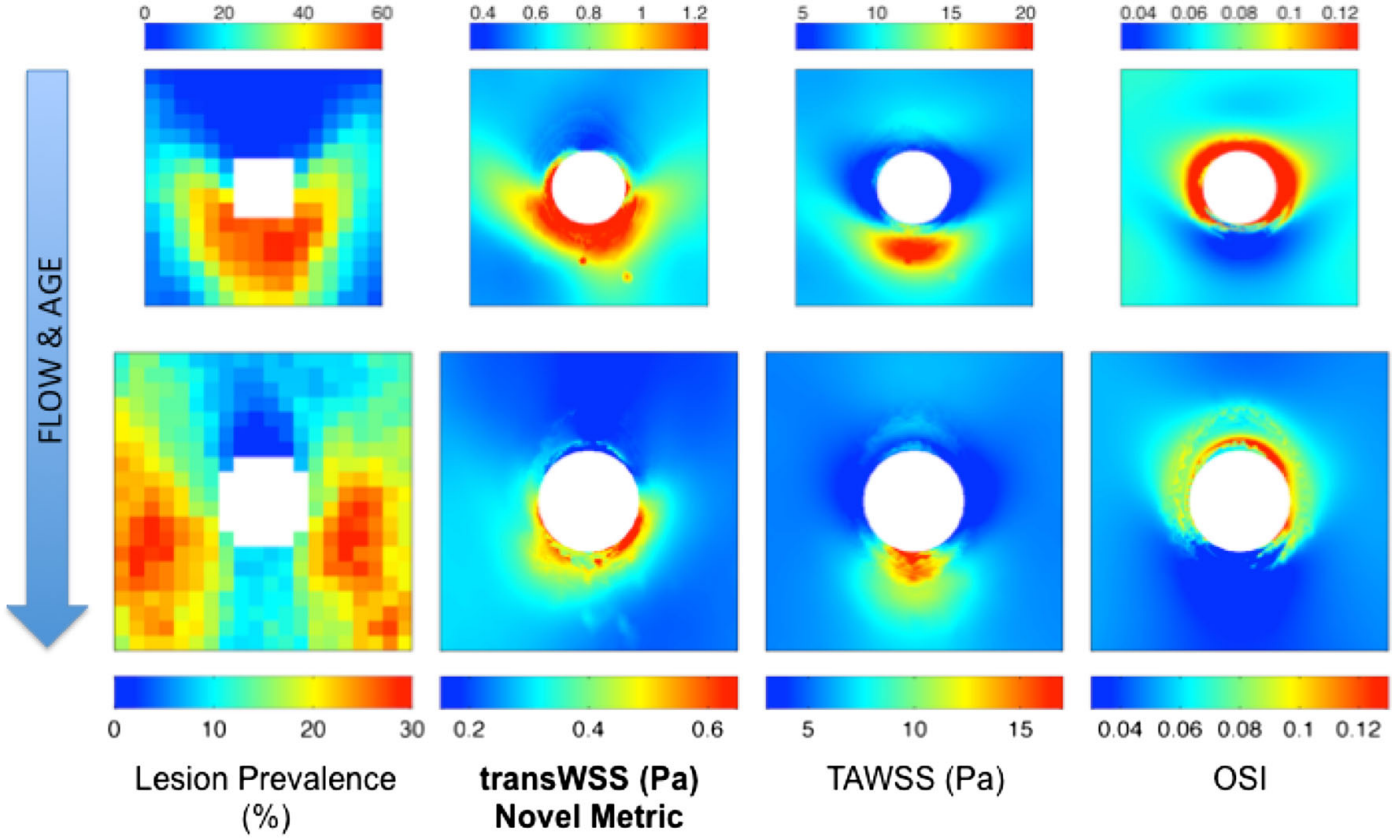
*En face* maps showing the pattern of lesion prevalence and three WSS metrics around intercostal branch ostia (white) in the aortas of immature (top row; 1.92 × 1.92 mm) and mature (bottom row; 2.4 × 2.4 mm) rabbits. Mean aortic flow is from top to bottom

**Figure 3:**
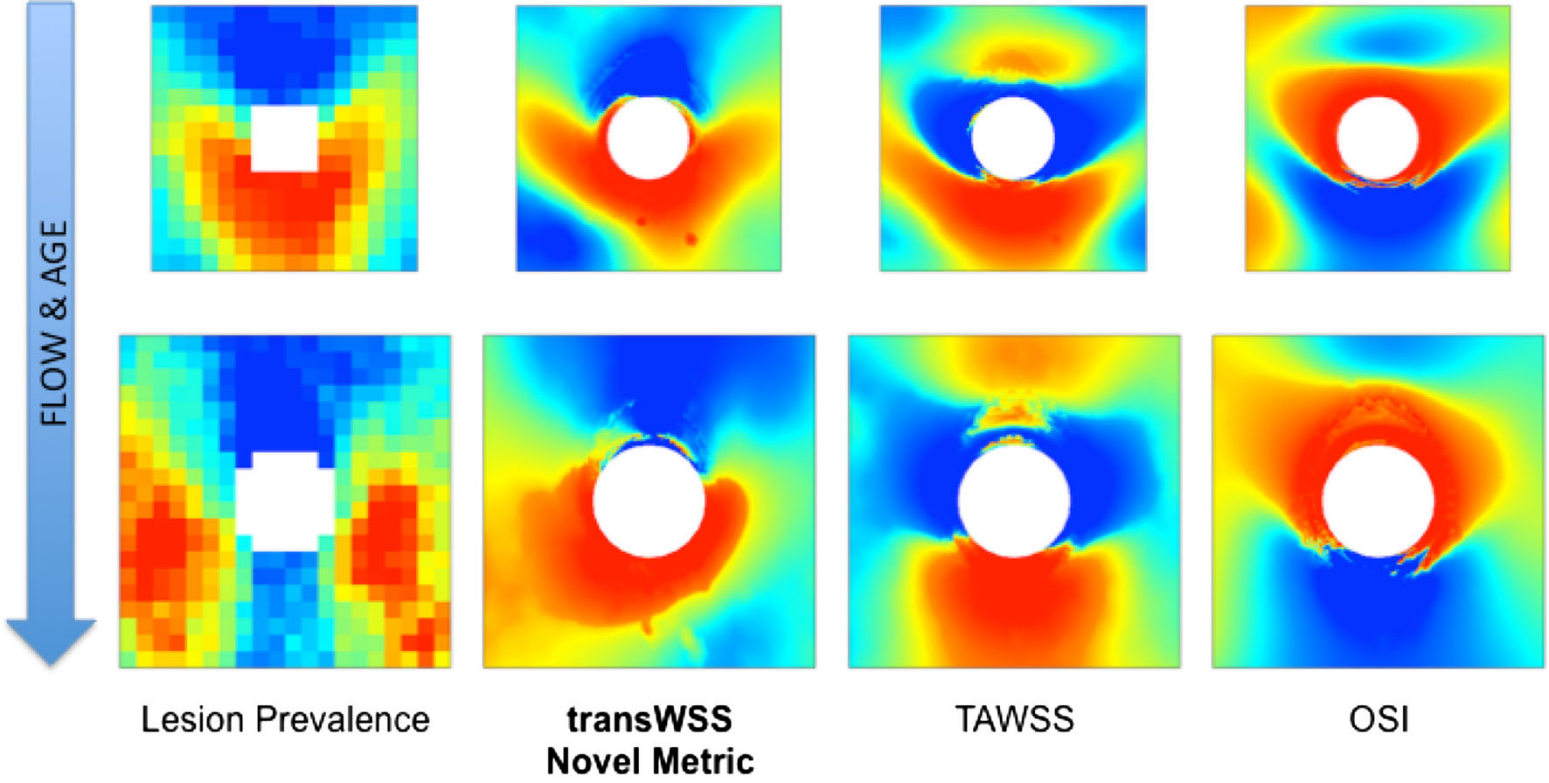
The maps shown in Fig. 2 but with absolute values replaced by the rank order of pixels (blue = lowest pixel intensity, red = highest pixel intensity)

Average maps of lesion prevalence and WSS metrics for immature and mature animals are shown in Fig. 2. The same maps are presented in Fig. 3 but absolute lesion frequencies and absolute values of WSS metrics have been replaced by ranks—that is, the pixel with the lowest intensity was given a rank of 1, the next lowest a rank of 2, *etc*.—to correspond with the ranking method used to calculate correlations between the maps.

In young animals, lesions occurred distal to branch ostia in the downstream arrowhead pattern first described by Anitschkow. In mature animals, the highest lesion prevalence occurred at the sides of the branches, with low prevalence upstream and downstream of the branch, along its centreline. This pattern has varied slightly between studies: the lateral peaks of high prevalence occurred more proximally in a meta-analysis^4^ of trials involving much older animals of the HSDIF strain fed a variety of atherogenic diets—if the ostium is considered as a compass rose then the lesions occurred at the “East” and “West” locations, rather than the “Southeast” and “Southwest” ones shown in Fig. 2—and the ratio of frequencies upstream and downstream of the branch is somewhat different in maps of the spontaneous lesions that occasionally occur in old rabbits.^3^ Nevertheless, in all cases the pattern is broadly similar to the one identified in young adult human aortas by Sloop *et al.* 33

Time average WSS in immature animals was elevated in a patch downstream of the branch, as predicted by the boundary layer arguments of Fry^14^ and Caro *et al.* 8 However it was also elevated, albeit to a lesser extent, upstream of the branch. That reflects the convergence of upstream streamlines caused by fluid entering the branch from regions lateral to it.^19^ The patches of elevated WSS had a slightly different shape in the mature animals, but the overall distribution did not change substantially.

A similar lack of fundamental change with age was seen close to the ostia in the maps of OSI. In both immature and mature animals, the highest OSI occured at the lateral and proximal margins of the ostium; only the balance between the lateral and proximal peaks changed slightly, the lateral ones being more accentuated in the immature animals. At both ages, values of the OSI were substantially lower further away from the ostium, and were lowest upstream and downstream of the branch.

The maps of transWSS showed the greatest change with age. In the immature animals, transWSS was high in an arrowhead-shaped pattern around the downstream half of the ostium. In the mature animals, however, the point and two barbs of the arrowhead largely disappeared, and the periostial region of high transWSS merged with longitudinal stripes of moderately elevated transWSS at the lateral margins of the map.

**Figure 4:**
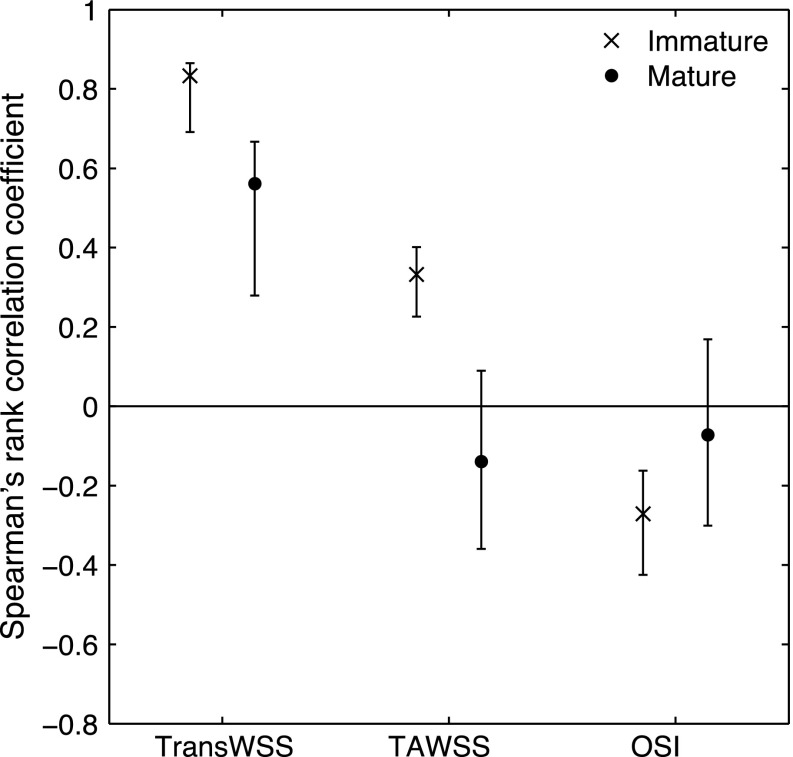
Mean correlation coefficients (with 95% confidence intervals) for the relation between lesion prevalence and the three WSS metrics shown in Figs. 2 and 3

Figure 4 shows the correlation coefficients and associated confidence intervals for the relation between lesion prevalence and the three shear metrics at both ages. Considering first the time average WSS, there was no significant correlation in the mature animals. There was a positive correlation in the immature animals. The correlation in the younger group was significant in this study despite being only borderline in our earlier investigation because the new statistical methods have somewhat greater power; however, the value of the coefficient was low. There was no significant correlation between lesion prevalence and the OSI for the mature animals, and a significant *inverse* correlation—albeit with another low coefficient—for the immature animals. TransWSS correlated significantly with lesion prevalence in mature animals, unlike the other two metrics. It also correlated significantly in the immature animals, and significantly more strongly than the other two metrics. At both ages, the correlation coefficients for the transWSS were positive and approximately three times those observed for the other metrics.

## Discussion

TransWSS correlated positively, strongly and significantly with lesion prevalence at both ages. Correlations of lesion prevalence with the other shear metrics were either not significant or significantly lower in magnitude than those obtained for transWSS. No correlations supported the low, oscillatory WSS theory. The data are consistent with the views that lesions in immature and mature aortas are triggered by the same type of hemodynamic stress, that the anatomical pattern of these stresses changes with age, and that multidirectional near-wall flow is highly atherogenic. These results are subject to some uncertainty. First, the mature pattern of lesions has varied slightly from study to study, although it retains the same essential characteristics.^3^,^4^,^10^ Second, the boundary conditions used in the numerical simulations are approximations; for example, Sloop *et al.* 33 showed that the aortic flow waveform near intercostal branch ostia changes with age in people, but such variations were not taken into account. Third, we only described results for a small region around branch ostia; the study demonstrated that in at least these areas correlations with the different shear metrics can be separated, and significantly stronger correlations obtained for transWSS, but it did not establish a universal connection. Additionally, we have not yet investigated a range of other shear metrics (including spatial and temporal WSS gradients) putatively linked to atherosclerosis. The likely significance (or otherwise) of multidirectional flow can be established further by studies that overcome these limitations but also by investigating effects of such flow on putatively pro- and anti-atherogenic behaviors of EC.

### Methods for Studying Effects of Transverse Flow *In Vitro*

We have employed two methods for studying effects of transverse or multidirectional flow on EC. One, developed by Wang *et al.*, uses the geometry of a standard parallel-plate flow chamber to accustom EC to flow from one direction. The EC, as is common in such apparatus, grow on a glass slide. In the new apparatus, however, this slide can be manually rotated by any angle in the plane of its surface. Since the flow direction remains constant, this results in the EC being exposed to flow from a direction other than the one to which they had adapted. The apparatus is described in more detail elsewhere.^35^

A strength of the method is that the flow is predictable and controllable. Numerical simulations and experiments examining particle tracks showed that the cells are exposed to uniform, laminar flow and a well-defined, steady shear stress.^35^ The change in angle of the flow can also be controlled precisely.

The second method is to grow EC in conventional circular dishes or multi-well plates placed on an orbital shaker. The orbit of the dish or well forces a swirling motion in the medium, and hence applies a shear stress to the cells. This method has been used for many years to compare effects of flow with no flow [e.g., Ref. 36]. However, it can also be used to distinguish effects of different types of flow because the temporal pattern of shear vectors varies with distance from the center of the well. Of particular interest in the current context is its potential to compare uniaxial and multidirectional flows.

Unfortunately, the flows are difficult to characterize. Experimental methods such as optical Doppler velocimetry have been used^12^ but most work, starting with that of Berson *et al.*,^5^ has employed numerical methods. The computational simulations are complicated by the presence of a free surface. Nevertheless, there is increasing understanding of the effects of well diameter, medium height, orbital radius and angular velocity on the spatial and temporal patterns of shear [e.g., Ref. 30]. At least one combination of these parameters gives a pattern of flow where all EC are exposed to a similar mean shear stress magnitude but shear vectors in the center of the well are more multidirectional than those near the rim.^28^ (However, excursions of shear magnitude from the mean are also greater near the rim.^28^)

A further disadvantage of the technique is the need to compare cell properties between different regions of the well, restricting studies to phenomena that can be measured in a spatially resolved way (usually by microscopical techniques). Its two substantial advantages are that the cells can be chronically exposed to the flows (which is more representative of the *in vivo* situation) and that throughput is high.

Ideal methods have yet to be devised. The first method would be improved if the multidirectional flows could be applied chronically, for example by a rotational oscillation of the glass slide, and if multiple slides could be used to increase throughput. Another possibility would be to alter the standard parallel-plate flow chamber in some other way, for example by incorporating multiple inflow and outflow ports. (Studies of this type have been conducted by Kataoka *et al.,* 18 but the frequency of change in flow direction was far removed from the physiological range considered here.) It might be possible to obtain more appropriate and better-controlled flows in the swirling plate method, and particularly to eliminate radial variation in the range of shears, by manufacturing non-cylindrical wells.

### Preliminary Data on Mechanisms

Despite the drawbacks of both techniques, interesting data have been obtained with them. We first consider measurements acquired by using the parallel plate device.^34^

Transient phosphorylation of the p65 subunit of the NF-κB transcription factor, a key trigger of inflammatory activation in EC, occurred 5 min after cells were turned 90° to the flow to which they had adapted for 24 h, but not if they were turned by 180° or 360°. (Phosphorylation activates the molecule). Phosphorylation of eNOS, generally regarded as atheroprotective, was not increased following rotation through 90°, but was increased by rotation through 180°. Phosphorylation of Akt, an upstream activator of eNOS, showed similar behavior to eNOS, except that the phosphorylation was maintained for longer.^34^

It appears that the polarity of the cells is what matters. These effects were not observed in EC that had been pre-exposed to flow for 2 rather than 24 h, and were therefore not aligned with that flow. Furthermore, when naïve EC were exposed to onset of flow, there was preferential nuclear translocation of NF-κB in individual cells that were oriented perpendicularly to the flow direction. Thus, orientation without flow modulates responses in the same way. Lastly, effects of oscillatory flow were examined on EC aligned on micropatterned fibronectin lines. Flow that was perpendicular to the cells’ direction stimulated reactive oxygen production and NF-κB activation whereas parallel flow had little effect. By contrast, phosphorylation of eNOS and production of NO were high in parallel and negligible in perpendicular flow. Thus oscillatory flow, widely regarded as pro-atherogenic, did indeed have pro-atherogenic effects when acting perpendicularly to aligned and elongated EC, but it had anti-atherogenic effects when acting parallel to them. This result may explain the absence of an overall correlation between the OSI and lesion prevalence described above.

An interesting response obtained by using the swirling plate system in conjunction with scanning ion conductance microscopy (SICM) was that EC cultured near the edge of the wells, where shear is most uniaxial, were more elongated and aligned and less compliant than cells in the center of the well, where the shear is multidirectional.^29^ When the SICM technique was applied to different regions of fresh porcine aorta, EC of the outer curvature of the arch, which is generally assumed to be protected from atherosclerosis, were more elongated and aligned and less compliant than cells from the atheroprone inner curvature.^29^ An association between high compliance and multidirectional flow *in vitro*, and with sites expected to develop lesions *in vivo*, is of interest because altered stiffness may represent an early event in mechanotransduction; EC become significantly more compliant as early as 30 s after exposure to shear stress.^11^

### Potential Upstream and Downstream Pathways

As we recently noted,^31^,^38^ a completely symmetrical cell cannot be sensitive to the direction of flow; some degree of elongation or functional asymmetry is required before transWSS can be recognized. This suggests that transWSS might have most effect where cells are more elongated. Why then, does purely oscillatory flow, where direction changes by 180°, activate NF-κB and induce inflammatory pathways in EC *in vitro*? (Low magnitudes of unidirectional flow, though less potent than oscillatory flow, also activate predominantly inflammatory pathways.^23^) Wang *et al.* 34 have suggested that the answer to this question may reside in the fact that low and oscillatory shears fail to induce EC alignment; the cells retain some elongation but are randomly oriented. Thus, even unidirectional or purely oscillatory flow will be perpendicular to a substantial fraction of the cells in the population. This complexity may account for seemingly contradictory reports of the association of EC shape with lesions.^6^,^15^ EC also align perpendicularly to the direction of cyclic stretch,^17^ so off-axis flow—and, by implication, high lesion prevalence—might also occur when the angle between flow and stretch is not equal to 90°.

Endothelial morphology could be directly involved in sensing transWSS. Flow over EC is locally perturbed because the surface of the cells is not flat, and the degree of perturbation depends on flow direction: peak WSS and peak WSS spatial gradient are both greater when flow is perpendicular to the long axis of aligned cells than when it is parallel.^2^ Internal determinants might also be involved. For example, shear direction could be sensed relative to cytoskeletal structures such as actin bundles, microtubules or focal adhesions, so long as these have a preferred orientation within the cell. Integrin and focal adhesion dynamics are already implicated in flow sensing, so are likely candidates. In this context, it is of interest that F-actin stress fibers are not always aligned with the long axis of the cell: Kim *et al.* 20 showed that their orientations can diverge by up to 30°.

Considering pathways further downstream, activation of NF-κB by transverse flow is likely to increase recruitment of circulating monocytes. The distribution of intimal white blood cells around the aorto-coeliac branch of rabbits bears little resemblance to maps of time average WSS or the OSI obtained from computational simulations of flow at the same branch, but does correspond to patterns of secondary flow visualized in physical models.^7^,^22^ The strength of the secondary flows varies during the cardiac cycle, so it would be of interest to examine the correspondence with transWSS. Finally, we note that uptake of plasma macromolecules by the rabbit aortic wall around intercostal branch ostia shows age-related patterns that visually resemble the patterns of transWSS and lesion prevalence^13^; we speculate that they depend on the former and help determine the latter.

## Conclusion

The new statistical investigation presented above showed that the different patterns of lesions seen around aortic branch ostia in immature and mature rabbit aortas both correlate significantly with the level of transWSS, an index of multidirectional flow. Further studies will examine a wider range of ages, the effect of varying boundary conditions, other WSS metrics that have been linked to atherosclerosis, and the correlations obtained in other parts of the arterial system. They will also determine why transWSS has an age-dependent pattern.

The correlation between transWSS and lesion prevalence observed at immature and mature rabbit intercostal branch points may be relevant to human disease since the two patterns of lipid deposition closely resemble those seen in very young^32^ and young adult^33^ human aortas. Together with the studies demonstrating possible mechanisms,^29^,^34^ the results have the potential, almost literally, to change the direction of the 50-year search for mechanical factors that localize atherosclerosis.

## Abbreviations

Akt: Protein kinase B
EC: Endothelial cells
eNOS: Endothelial nitric oxide synthesis
$\vec{n}$: The normal to the arterial surface
NF-κB: Nuclear factor kappa-light-chain-enhancer of activated B cells
NO: Nitric oxide
OSI: Oscillatory shear index
SICM: Scanning ion conductance microscopy
*t*: Time
*T*: The period of the cardiac cycle
TAWSS: Time average wall shear stress
transWSS: Transverse wall shear stress
WSS: Wall shear stress
